# Exploring Quantum Neural Networks for Demand Forecasting

**DOI:** 10.3390/e27050490

**Published:** 2025-05-01

**Authors:** Gleydson Fernandes de Jesus, Maria Heloísa Fraga da Silva, Otto Menegasso Pires, Lucas Cruz da Silva, Clebson dos Santos Cruz, Valéria Loureiro da Silva

**Affiliations:** 1 QuIIN–Quantum Industrial Innovation, EMBRAPII CIMATEC Competence Center in Quantum Technologies, SENAI CIMATEC, Av. Orlando Gomes, Salvador 41650-010, Bahia, Brazil; otto.pires@fieb.org.br (O.M.P.); valeria.dasilva@fieb.org.br (V.L.d.S.); 2 Latin America Quantum Computing Center, SENAI CIMATEC, Salvador 41650-010, Bahia, Brazil; 3 Grupo de Informação Quântica e Física Estatística, Centro de Ciências Exatas e das Tecnologias, Universidade Federal do Oeste da Bahia, Barreiras 47810-059, Bahia, Brazil; clebson.cruz@ufob.edu.br; 4 Robotics Department, SENAI CIMATEC, Salvador 41650-010, Bahia, Brazil; lucas.cs@fieb.org.br

**Keywords:** demand forecasting, quantum machine learning, quantum finance, vehicle financing, predictive modeling

## Abstract

Forecasting demand for assets and services can be addressed in various markets, providing a competitive advantage when the predictive models used demonstrate high accuracy. However, the training of machine learning models incurs high computational costs, which may limit the training of prediction models based on available computational capacity. In this context, this paper presents an approach for training demand prediction models using quantum neural networks. For this purpose, a quantum neural network was used to forecast demand for vehicle financing. A classical recurrent neural network was used to compare the results, and they show a similar predictive capacity between the classical and quantum models, with the advantage of using a lower number of training parameters and also converging in fewer steps. Utilizing quantum computing techniques offers a promising solution to overcome the limitations of traditional machine learning approaches in training predictive models for complex market dynamics.

## 1. Introduction

A common problem experienced by companies is financial market uncertainty [1,2], which makes accurate forecasting and budgeting challenging, becoming a risk to investments and financial stability [1,2,3,4]. Companies must adapt quickly to these changes in order to remain competitive and mitigate any negative impacts [5]. In this context, demand forecasting is defined as a predictive analysis strategy used to overcome this challenge using traditional computational methods or more advanced technologies, including machine learning [6,7,8].

In this scenario, the demand estimation process helps companies internally plan to meet market needs [8,9]. It involves forecasting the number of services or products a company will sell in a future period [10]. The duration of this period can be customized and may vary based on the company’s size and objectives [11].

In order to help managers make more assertive decisions about team planning and demand management, forecasting takes into account internal and external factors that meet customer needs [12]. The benefits encompass enhancements in efficiency, operational performance, and the supply chain as it predicts the number of goods to be sold and, subsequently, the amount that has to be manufactured [13], thereby preventing the occurrence of insufficient or excessive production. Quantitatively, if a bank with an annual vehicle financing volume of USD 10 billion, for example, uses a predictive model that results in a 10% loss in potential revenue, an improvement in accuracy that reduces this rate to 5% could represent an additional gain of USD 500 million annually.

Furthermore, demand forecasting can be influenced by either a qualitative or quantitative methodology [14]. The first case is a superficial subjective analysis of customer behavior and market trends. In the second case, statistical data are compared and analyzed from both the sales history and customer base to provide a more in-depth picture of the future [6]. Accordingly, demand forecasting was traditionally based on statistical methods and expert opinion, which often made it difficult to capture complex patterns and dynamic market trends [15]. Given this scenario, the implementation of machine learning algorithms in demand forecasting has resulted in notable improvements in forecast accuracy [15].

In this context, classical machine learning (ML) is a widely used computational tool for solving the problem of demand forecasting [16]. It helps identify patterns in large volumes of historical data and make accurate forecasts. However, as data volumes increase and models become more complex, the processing limitations of classical models become more apparent due to the difficulty of capturing multiple characteristics of high-dimensional data [17]. Conversely, quantum computing has emerged as a promising solution. The ability of quantum computers to process information in parallel offers a significant advantage, allowing for the efficient and quick analysis of massive datasets and the optimization of machine learning models [18,19].

Thus, *quantum machine learning* (QML) emerges as a promising alternative that can accelerate information processing and provide notable improvements in the machine learning research area [19,20]. Recent advancements in this area suggest that the integration of quantum computing with machine learning is poised to lead to groundbreaking developments in technology and data analysis [21,22]. This approach offers a way to address the difficulties presented by conventional machine learning techniques [22,23], such as increased learning duration caused by the expanding amount of data [24]. Hence, quantum computing and quantum machine learning (QML) have recently experienced increased utilization across various domains, including finance [25].

In this regard, this work presents an application for QML to predict vehicle financing demand using a quantum neural network. For this purpose, we utilized a dataset obtained from the Brazilian bank BV, containing financing data and other relevant features collected from 2019 to 2023. These data were pre-processed, and smaller sets were extracted using feature reduction techniques [26,27]. The quantum neural network was trained on these pre-processed data to accurately predict vehicle financing demand, showcasing the potential of quantum computing in enhancing predictive analytics. The results obtained from this study demonstrate the promising capabilities of QML models in solving complex real-world problems such as financial forecasting. The integration of quantum computing in predictive analytics can revolutionize the way financial institutions make decisions and manage risks. By leveraging the power of QML, banks can gain a competitive edge in the market by making more accurate and timely predictions. Therefore, this research highlights the importance of leveraging quantum computing in the financial sector to improve decision-making processes, which represents a significant advancement in the field of predictive analytics.

## 2. Quantum Neural Networks

Quantum neural networks (QNNs) represent a new approach to machine learning, combining classical data processing with the power of quantum computing [19,20,28,29]. Despite their classical foundations, QNNs are considered *pure quantum* models because their execution depends on classical computing only for circuit preparation and statistical analysis [30]. These QNNs fall under Variational Quantum Algorithms, employing Parameterized Quantum Circuits (PQCs) known as *ansätze* (plural of ansatz), which are trained using classical optimization techniques. The behavior of quantum neural networks (QNNs) reflects that of classical neural networks, consisting of three main stages: data preparation, data processing, and data output [30].

In the data preparation stage, the classical input is encoded into a quantum state using a feature map, a circuit parameterized exclusively by the original data [31]. This coding facilitates the integration of the classical information into the quantum structure of the QNN. In particular, classical data may require pre-processing, such as normalization or scaling, to optimize the coding process [31,32].

**Figure 1:**
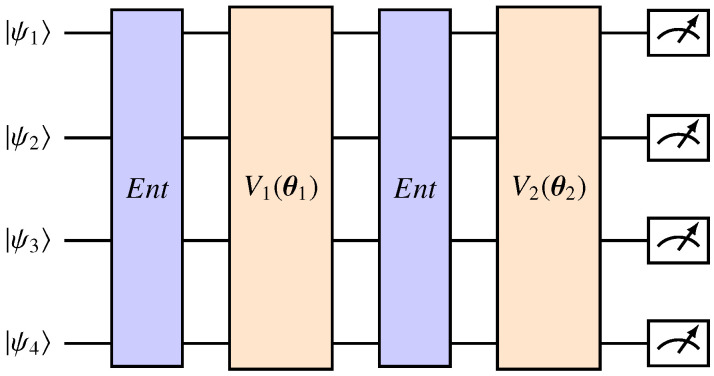
A two-layered ansatz applied to four qubits. Each layer is defined by a variational circuit $V_{j}$ dependent on some parameters $\theta_{j}$. The circuits Ent are used to entangle the qubits, and the state ${|\psi \rangle }^{⊗n}$ denotes the output of the feature map.

Subsequently, in the data processing stage, the QNN operates within the framework of its ansatz. Usually structured as a layered variational circuit, the ansatz consists of multiple layers, each defined by an independent parameter vector. Variational circuits $V_{j}$ dependent on these parameters make up each layer, with layers of entanglement Ent interspersed. An example of a quantum ansatz is shown in Figure 1. The ansatz effectively processes quantum-coded data, taking advantage of entanglement and parameterized gates for computation.

Finally, in the data output stage, the processed quantum state is converted into a classical output via a final layer [30]. This operation is adapted to the specific problem being addressed. For example, in a binary classification, the expected value of a single qubit selected in the measurement can be used as the output [31]. Overall, QNNs offer a promising path for quantum-assisted machine learning, uniting classical and quantum paradigms to address complex computational tasks [19,20,31,32].

In this article, we use a QNN, whose architecture is described in Section 3.2, to perform the task of forecasting the demand for used vehicle financing in Brazil.

## 3. Quantum Data Analysis and Model Implementation

### 3.1. Data Scaling and Selection Techniques

The case analyzed in this paper was the forecasting of used car prices in Brazil from May 2022 to April 2023. The data used for training covered the period from January 2019 to April 2022, and they were provided by the Brazilian bank BV, so the application is of practical interest in the financial sector. In all, 25 features were provided for the training. However, 6 features were discarded because they did not contain data for 2019. The remaining 19 features were subjected to a feature reduction process using the Principal Component Analysis (PCA) so that the features with the greatest variance were selected. PCA is a statistical technique that enables the reduction of data dimensionality while preserving variance. It identifies principal components, linear combinations of initial features, ranked based on their total variance, ensuring that significant new features are identified [26].

**Figure 2:**
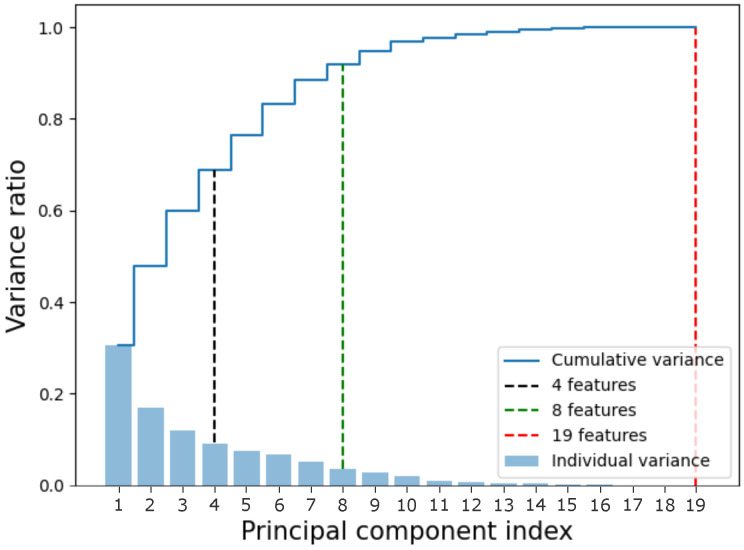
Cumulative variance of the data. The x-axis represents the component index, while the y-axis represents the variance. The sets used in this article were marked in black (4 features), green (8 features), and red (19 features, the complete dataset). The bars represent the individual variance of each component, while the blue line represents the cumulative variance.

The smallest sets used had 4 and 8 features, which represented 68.69% and 92.03% of the total variance of the dataset, respectively. In addition, the complete dataset represents 100% of the total variance of the distribution. The cumulative variances are shown in Figure 2.

After reducing the number of features, the data were standardized according to the following expression [32]:

(1) $$\hat{x}=\frac{x-\mu }{\sigma },$$

where *x* is the original data, μ is the mean of the values, σ is the standard deviation, and $\hat{x}$ is the standardized data. This standardization assumes that the distribution of the data is approximately normal. Standardizing the data helps to ensure that all variables are on the same scale, which is important for many machine learning algorithms since it reduces the scale of the dataset, thereby decreasing the differences in scale between features and avoiding biases in features with larger scales. This process makes it easier to compare and interpret the coefficients of different features in the model.

### 3.2. Quantum Neural Network Architecture

**Figure 3:**
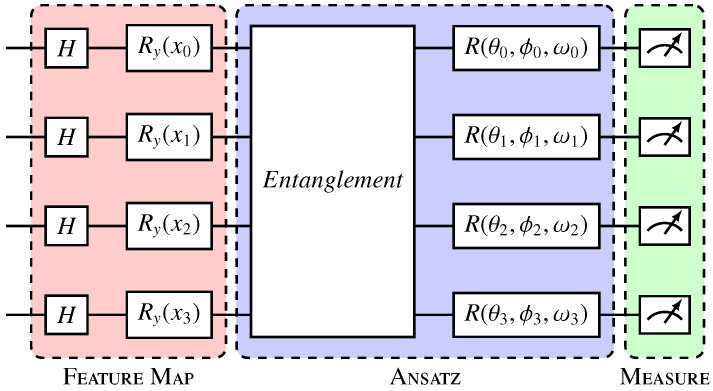
Variational quantum circuit. The Hadamard gate layer prepares the qubits in uniform superposition; Ry gates (red) encode the data in qubits; and the variational layer or ansatz (blue) entangles the qubits and applies parameterized rotations, where $\theta_{i}$, $\phi_{i}$, and $\omega_{i}$ represent, respectively, the rotation angles in the x, y, and z axes in each qubit *i*, and are the trainable parameters of the model. The measurement layer (green) collapses the qubits, generating the outputs [32].

**Figure 4:**
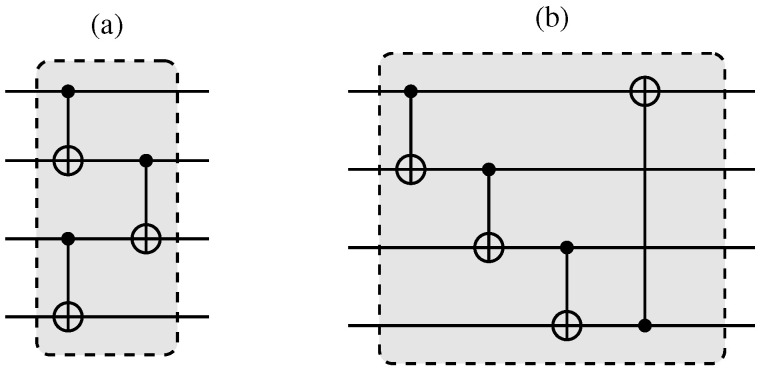
Entanglement layers used in a variational circuit (Figure 3). In (**a**), here named “entanglement layer 1”, the qubits are entangled in pairs, and these pairs are subsequently tied together. In (**b**), here named “entanglement layer 2”, the qubits are entangled in a cascade. Adapted from [32].

In quantum neural network architecture, qubits are used to represent data and parameters in the model [33]. By leveraging quantum superposition and entanglement, quantum neural networks have the potential to outperform classical neural networks in certain tasks by processing information in a more efficient way [30,34]. This architecture holds promise for solving complex problems in fields such as optimization, machine learning, and cryptography. The quantum neural network model used to process these data is presented in Figure 3. In this model, we used Angle Embedding with RY rotation gates as our feature map, performed after initializing the circuits in uniform superposition through Hadamard gates. Due to the reduced number of features in our datasets, still within the range of a classical simulator, Angle Embedding has the advantage of low cost compared with other embeddings, only a single layer of single qubit operations. Two variational layers were considered and are represented in Figure 4.

In order to take into account the real monetary losses due to inaccurate results, the models were analyzed based on the mean absolute error obtained in each experiment. In addition, the accuracy of the results obtained through the heuristics used was analyzed by calculating the standard deviation obtained during the 12 months of testing. Each experiment was run 10 times.

## 4. Dataset and Preprocessing

In addition to the variable to be predicted, the dataset made available for the research contained 25 economic features relevant to the proposed model, with 52 samples collected monthly from January 2019 to April 2023. The model’s prediction variable is the daily average of used vehicle financing each month and was also part of the dataset made available.

During the period covered in the database, the world faced the COVID-19 pandemic, and governments around the world shut down their countries’ economies to promote social isolation. For this reason, we can consider this period to be anomalous.

Training the model in more predictable environments could help with the training process, potentially leading to better results. However, removing the COVID data would mean reducing the already scarce number of samples available for training, so they have been kept in their original form. In addition, as it is understood that not the pandemic itself but its impacts on economic indicators (contained in the database used) are the most relevant features for the predictive model in question; no new features were added to the database that could bring new information about the pandemic scenario. It should also be considered that the proposed model must be robust to any factors that impact the financial market, and the inability to predict such occurrences makes it necessary to measure them indirectly through their influence on economic indicators.

When analyzing the data made available for the research, it was noticed that some features did not contain data for 2019. As the number of instances for training was already low, it was necessary to remove these features since the alternatives to such exclusion would be to exclude the data for the entire year 2019 or to infer the missing data, which could make the model biased and was therefore not done. After excluding the 6 features that did not contain data for 2019, a feature reduction was carried out using the PCA method [26,27].

In addition, the PCA method allows for the reduction of the number of features in a dataset by applying a transformation to the coordinate axes. This transformation generates new axes that point in the directions of the greatest variance in the datasets. The directions of greatest variance are the main components of the models since they supposedly have more information to extract during the training process and can then be used in this process. The PCA method was used through the scikit-learn [35] machine learning library.

Based on the data available and after eliminating the data that did not have values for 2019, 3 different sets of data were generated, with 4, 8, and 19 features, which represent 68.89%, 92.03%, and 100% of the system’s total variance, respectively. These sets were generated to assess the impact of adding new features to the models.

## 5. Results

The results obtained from the quantum experiments are presented in Section 5.1, while those from the classical experiments are detailed in Section 5.2. The number of variational layers selected for the quantum experiments includes configurations of 1, 3, and 5 layers, chosen to assess their impact on performance.

The measurement results are presented in terms of the distributions observed. Training and test errors are illustrated graphically, focusing on the average daily financing obtained. Additionally, the mean monthly absolute errors are provided in tables, offering a comprehensive view of the variations and trends.

### 5.1. Quantum Experiments

#### 5.1.1. Four Features

In the first considered case, the dataset was reduced from 19 initial features to 4 features. Similar to the other quantum experiments, we considered the two quantum networks presented in Section 3, as well as a classical RNN.

**Figure 5:**
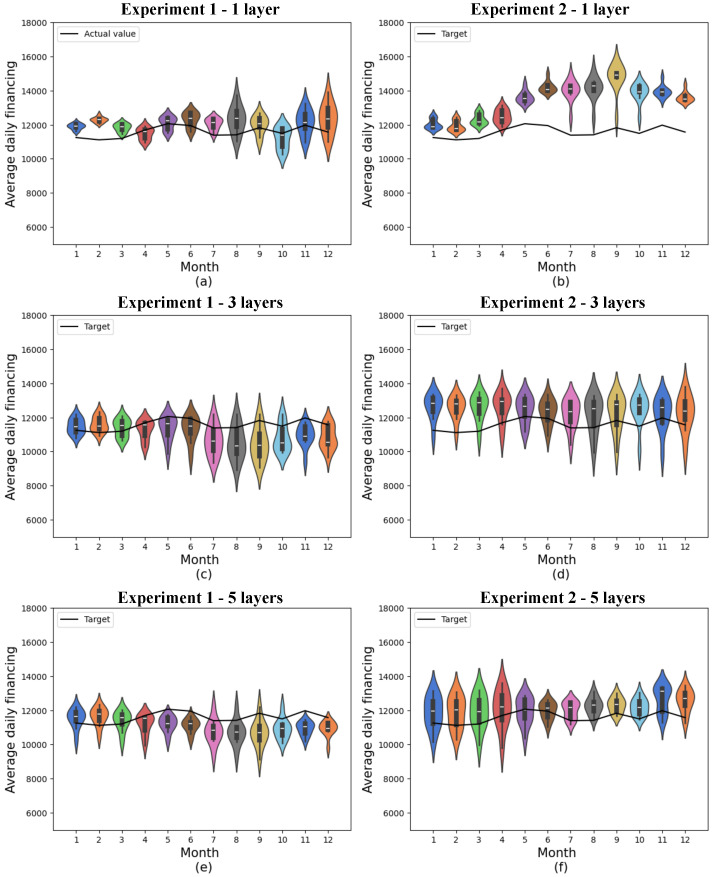
Predictions for quantum models with 4 features. (**a**) shows quantum experiment 1 with 1 layer, (**b**) quantum experiment 2 with 1 layer, (**c**) quantum experiment 1 with 3 layers, (**d**) quantum experiment 2 with 3 layers, (**e**) quantum experiment 1 with 5 layers, and (**f**) quantum experiment 2 with 5 layers. The x-axis shows the model’s training months, while the y-axis represents average daily financing. The distributions obtained from 10 experiments are shown in the colored violin graphs, while the actual values are shown in the black line.

Figure 5 shows the results of the quantum networks obtained in the two experiments using the 4-feature dataset and varying the number of variational layers. The actual values to which the predictions should approximate are shown in the black curves. The simulation environment is discussed in Section 5.3, and the convergence of the models is discussed in Section 5.5. The standard deviation for each month is presented in Table A1, in Appendix B.1, and the monthly mean absolute error for the two experiments is presented in Table A5, in Appendix C.1. The cumulative variance of the data contained in this database concerning the original database with 19 features is 68.69%.

Violin graphs were used to present the results of the predictions. These graphs show the density distributions through their contours. In this way, wider points in the figures represent a greater density of data, while sparser and more distant points represent outliers. In addition, these graphs can reveal multimodal trends in the distributions when there is more than one widening point. The black boxplot in the center of the figures shows the median of the distributions through a white line in the boxes, and the first and third quartiles are represented respectively through the lower and upper edges of the boxplot.

Considering realistic scenarios where errors are inevitable, it may be preferable to err upwards or downwards, depending on the market and the agents involved. Nonetheless, it is important to consider that errors above the target may be a warning of the need for greater production of a certain product or availability of services, while errors below the target may represent missed opportunities to sell products or services with a higher demand than predicted by the machine learning models.

Among the quantum models consisting of 4 features, experiment 1, involving a single variational layer, generally showed the best accuracy, with a minimum mean absolute error of 299.90 and a 12-month mean absolute error of 682.52 ± 284.14.

In the second experiment, the best result was the one in which 5 variational layers were considered, where the lowest mean absolute error obtained was 411.97, and the monthly mean was 785.98 ± 192.21. In addition, this model shows considerably less variation than the results obtained with just 1 variational layer, as well as a considerably lower mean.

Furthermore, in both cases, the means of the predictions were above the actual values, which could imply that there is a greater supply of used car finance than there is actual demand if these indicators are the only ones considered.

The results obtained from experiment 2 with 1 layer consistently exceeded the target at all times, deviating from the target especially in the final months. This outcome indicates a lower performance of this model in comparison with the other experiments with the same number of features.

#### 5.1.2. Eight Features

In the second considered case, the number of features in the database was reduced to 8. This value was chosen as an intermediate value between the 4 features used initially and the final 19 features, given that the cumulative variance for 8 features was 92.03%.

**Figure 6:**
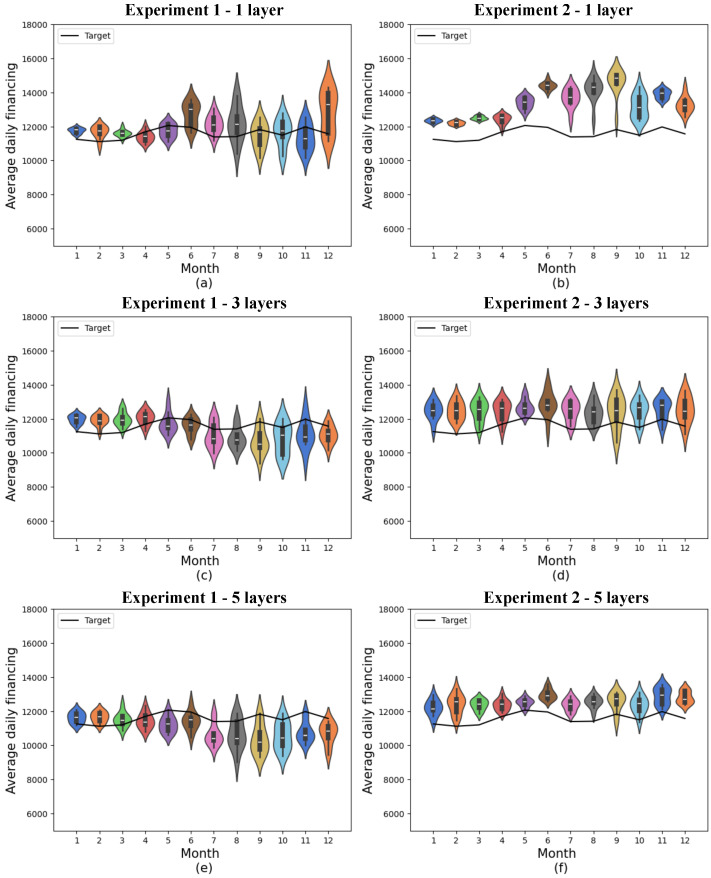
Predictions for quantum models with 8 features. (**a**) shows quantum experiment 1 with 1 layer, (**b**) quantum experiment 2 with 1 layer, (**c**) quantum experiment 1 with 3 layers, (**d**) quantum experiment 2 with 3 layers, (**e**) quantum experiment 1 with 5 layers, and (**f**) quantum experiment 2 with 5 layers. The x-axis shows the model’s training months, while the y-axis represents average daily financing. The distributions obtained from 10 experiments are shown in the colored violin graphs, while the actual values are shown in the black line.

The distributions obtained are shown in Figure 6. The standard deviation for each month is shown in Table A2, and the monthly mean absolute error for the two experiments is shown in Table A6. The cumulative variance of the data contained in this database concerning the original database with 19 features is 68.69%.

In the quantum models containing 8 features, experiment 1 with a single variational layer generally showed the best accuracy, with a minimum mean absolute error of 346.10 and a 12-month mean absolute error of 709.76 ± 297.93.

In the second experiment, the best result was again the one in which 5 variational layers were considered, where the lowest mean absolute error obtained was 441.65, and the monthly mean was 980.79 ± 239.93. As occurred in the experiment with 4 features, there is no statistical difference between this result and the result obtained with 3 variational layers. However, the results of this model showed considerably less variation than the results obtained with just 1 variational layer and also a considerably lower mean.

In this case, the forecast means were more distributed compared with the target in the best result of experiment 1, but the results of the second experiment echoed the trend of exceeding the actual values, generating a production signal above actual demand.

As observed in the experiment with 4 features, in the experiment with 8 features, the results obtained from experiment 2 with 1 layer remained above the target at all times, deviating from the target in the final months. This indicates a lower performance of this model in comparison with the others.

#### 5.1.3. Nineteen Features

In the third considered case, the dataset was tested using all the features for which data were available for 2019. Features that did not have data for 2019 were discarded. The alternative to discarding these features would be to perform inference for 2019 data. However, the amount of data that would be inferred would represent approximately 1/4 of the dataset, a portion that would compromise the model’s performance.

In order to train the model with this dataset, no transformation other than data standardization was carried out. However, from the 8th month of the test set onwards, it was identified that one of the features had increased significantly in relation to the others. For this reason, it was decided to maintain these data to observe the effects that this increase in one of the features would have on the results.

**Figure 7:**
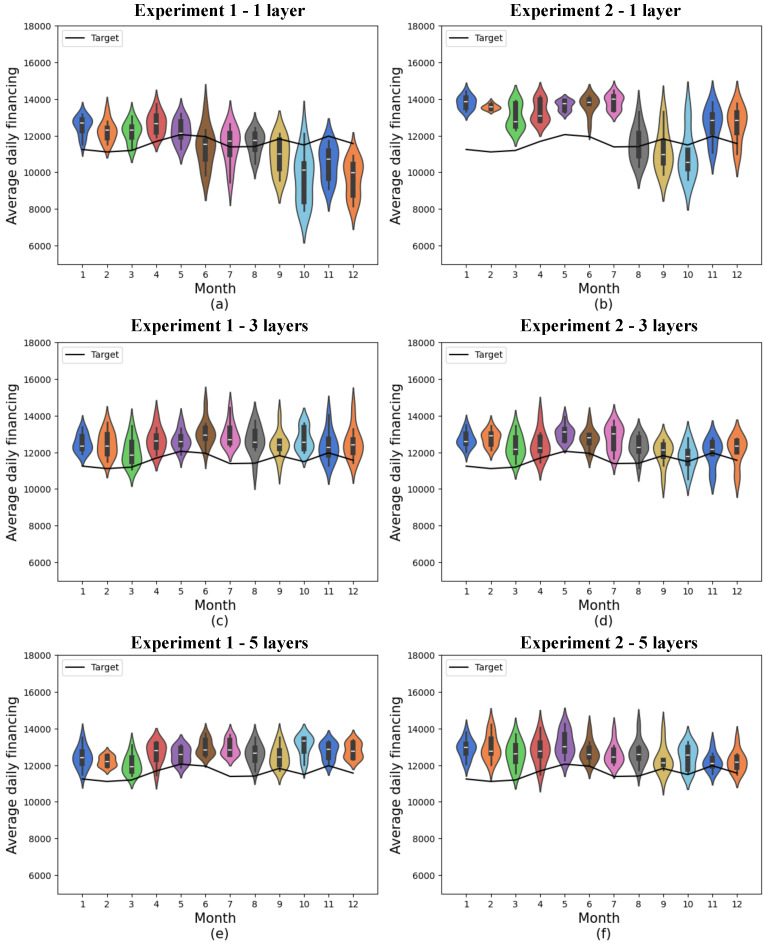
Predictions for quantum models with 19 features. (**a**) shows quantum experiment 1 with 1 layer, (**b**) quantum experiment 2 with 1 layer, (**c**) quantum experiment 1 with 3 layers, (**d**) quantum experiment 2 with 3 layers, (**e**) quantum experiment 1 with 5 layers, and (**f**) quantum experiment 2 with 5 layers. The x-axis shows the model’s training months, while the y-axis represents average daily financing. The distributions obtained from 10 experiments are shown in the colored violin graphs, while the actual values are shown in the black line.

The distributions obtained are shown in the subfigures provided in Figure 7. The standard deviation for each month is shown in Table A3, and the monthly and annual mean absolute errors for the two experiments are shown in Table A7. Since this database contains all the features from the original dataset, the cumulative variance of the data contained in this database is the total variance of the original set, i.e., 100%.

In experiment 1, which was performed with 19 features, there was no significant difference between the results obtained with 1, 3, and 5 variational layers. For all three cases, the minimum absolute mean errors obtained were 522.95, 638.46, and 533.07, and the 12-month means were 1141.58 ± 465.98, 1054.52 ± 298.52, and 1051.39 ± 311.39, respectively. In experiment 2, the smallest mean absolute monthly errors were 758.83, 552.07, and 365.82, respectively, and the monthly means were 1630.98 ± 625.66, 995.71 ± 374.16, and 1074.90 ± 450.09. In this case, however, the first result presents a larger error linked to a larger standard deviation.

Once again, the results obtained from experiment 2 with 1 layer performed worse than the others, indicating that the model exhibited lower performance.

### 5.2. Classical Experiments

**Figure 8:**
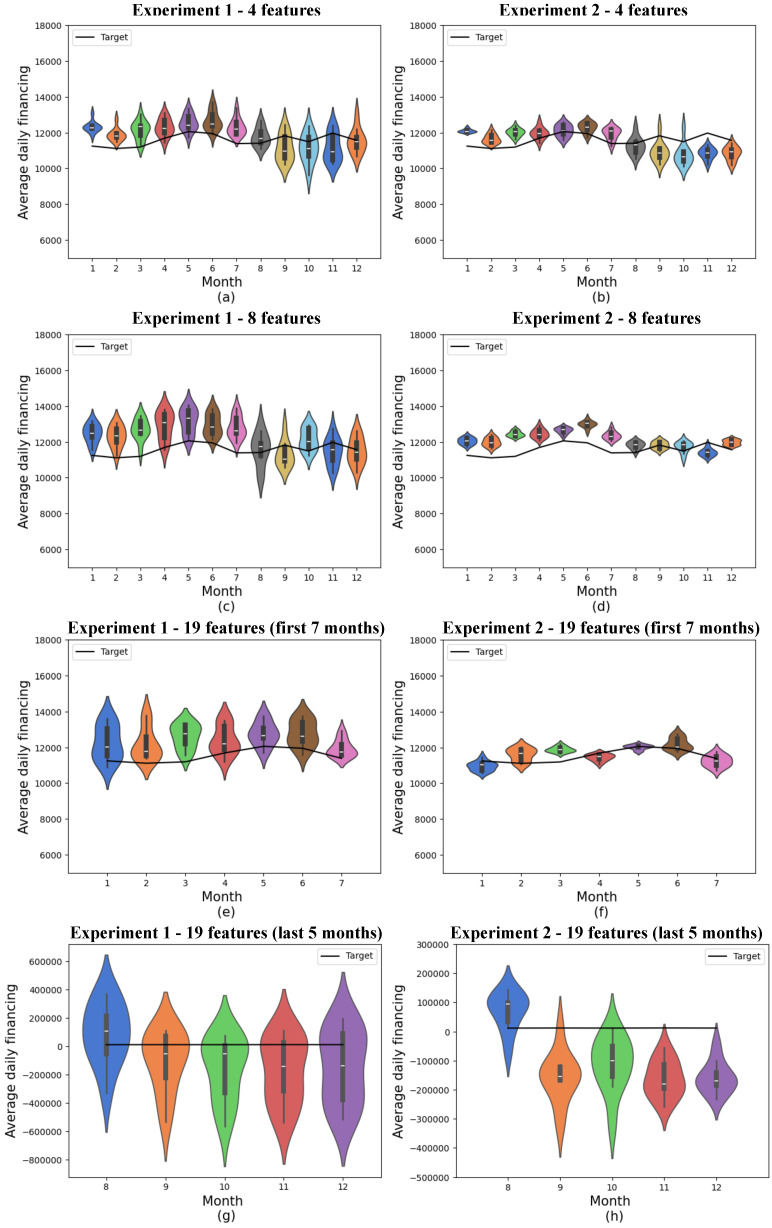
Predictions for classical models. (**a**) shows classical experiment 1 with 4 features, (**b**) classical experiment 2 with 4 features, (**c**) classical experiment 1 with 8 features, (**d**) classical experiment 2 with 8 features, (**e**) the first 7 months of classical experiment 1 with 19 features, (**f**) the first 7 months of classical experiment 2 with 19 features, (**g**) the last 5 months of classical experiment 1 with 19 features, and (**h**) the last 5 months of classical experiment 2 with 19 features. The x-axis shows the model’s training months, while the y-axis represents average daily financing. The distributions obtained from 10 experiments are shown in the colored violin graphs, while the actual values are shown in the black line.

The classical experiments were performed as a way of comparing the results obtained with those of traditionally used classical methods. For this purpose, a classical recurrent neural network with 128 and 1024 neurons in the recurrent layer was considered. RNNs were selected for benchmarking because they are a well-established method for time series forecasting, provide a robust benchmark for comparison, and help evaluate the potential benefits of quantum neural networks in demand prediction, as they are capable of using temporal correlation in the data to make predictions. Figure 8 shows the results obtained using this model, and convergence graphs are shown in Section 5.5.

In experiment 1, involving 4 and 8 features, the results were similar, so the 12-month means of the mean absolute errors were 774.93 ± 199.39 and 997.12 ± 331.18, respectively. However, when all 19 initial features were taken into account, these results were skewed by the variable that had significantly higher values in the last 5 months, so the error increased significantly, and the 12-month mean was 90,480.42 ± 111,210.54. Given that this increase in error and error variance only occurs in the last few months of training, the results for 19 characteristics are presented in two graphs, as the results scale has changed.

Disregarding the last 5 months, the model showed a mean absolute error of 344.19 with a mean standard deviation of 197.55, which is significantly better than all the other models (classical or quantum) presented.

In experiment 2, involving 4 and 8 features, the results also behaved similarly so that the 12-month means of the absolute mean errors were 643.12 ± 293.77 and 683.30 ± 332.37. As was the case in the first experiment, when all 19 initial features were considered, these results were biased by the variable that had significantly higher values in the last 5 months so that the error increased significantly, and the 12-month mean in this experiment was 7965.14 ± 61,675.02. The results of this experiment, considering 19 features, were also presented in two graphs since the scale of the results was modified.

Table 1 summarizes best results of classical and quantum annual mean MAE according to the results above and the tables in Appendix B.

### 5.3. Simulation Environment

All the simulations were performed in the PennyLane quantum computing software development kit [36], developed by the quantum computing company Xanadu [37], also using the TensorFlow machine learning library [38]. The simulations were carried out in an HPC environment on Intel Xeon Platinum 8260L processors. The simulations involving 19 features were performed on 17 cores, while the simulations involving 4 and 8 features were performed on a single core.

### 5.4. Training Time

The execution times for each sample of the quantum model are shown in Table 2, and for the classical model, in Table 3. The total computing time of the runs is given by the values in the table multiplied by ten since ten samples were extracted for each model.

Given that the demand forecasting problem was solved through a simulation and not on a quantum computer, it is not possible to correlate the processing times obtained with those obtained on a quantum computer. Simulating quantum circuits is an expensive simulation that uses an exponential number of resources in relation to the number of qubits. This demand for resources would scale linearly on a quantum computer, since they can naturally implement the quantum properties that must be simulated through classical algorithms. Instead, the rapid convergence of the models can be interpreted as an indication of shorter execution times required by the quantum model.

Processing time is often pointed out as an advantage of quantum computing since some quantum algorithms have an advantage over the best classical algorithms that perform the same task. For instance, Shor’s algorithm and Grover’s algorithm are able to perform tasks in exponentially and quadratically less time than a classic computer, respectively. However, when analyzing the computational advantages, other metrics must be taken into account, such as the accuracy of the results and the savings in computational and energy resources.

### 5.5. Convergence

**Figure 9:**
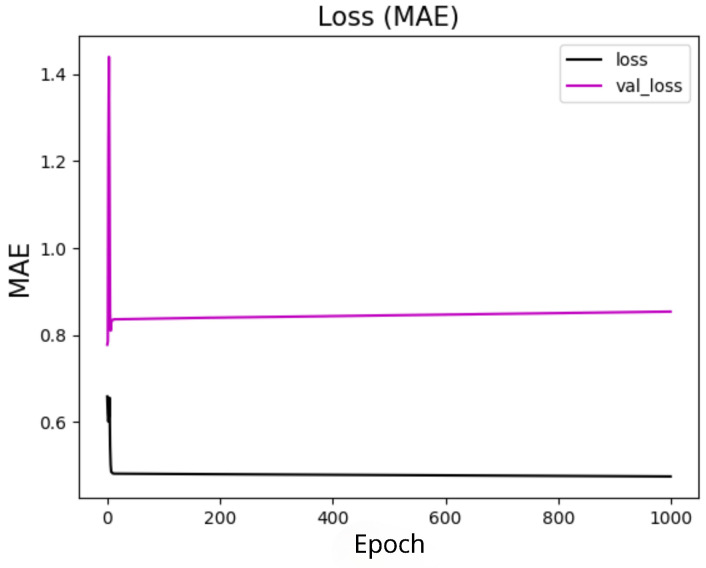
Convergence of the quantum model with the set of 4 features and 1 layer with 1000 epochs. The x-axis shows the training epochs, while the y-axis shows the mean absolute error (standardized values). The black curve shows the test loss, while the magenta curve shows the validation loss.

**Figure 10:**
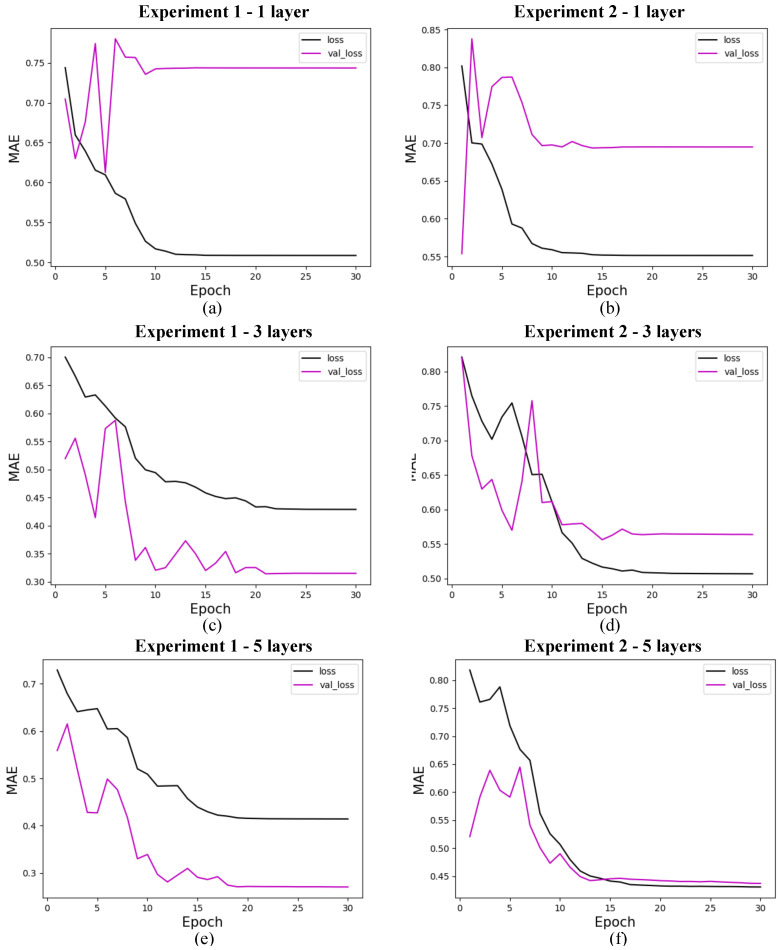
Loss for quantum models with 4 features. (**a**) shows the loss for quantum experiment 1 with 1 layer, (**b**) for quantum experiment 2 with 1 layer, (**c**) for quantum experiment 1 with 3 layers, (**d**) for quantum experiment 2 with 3 layers, (**e**) for quantum experiment 1 with 5 layers, and (**f**) for quantum experiment 2 with 5 layers. The x-axis shows the training epochs, while the y-axis shows the mean absolute error (standardized values). The black curve shows the test loss, while the magenta curve shows the validation loss.

**Figure 11:**
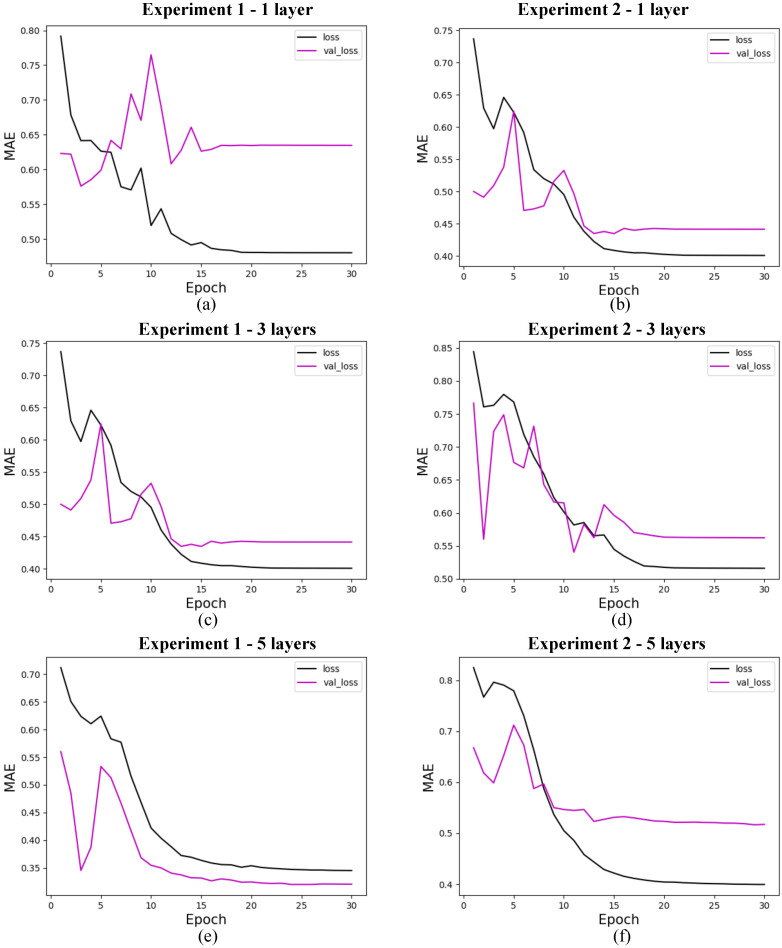
Loss for quantum models with 8 features. (**a**) shows the loss for quantum experiment 1 with 1 layer, (**b**) for quantum experiment 2 with 1 layer, (**c**) for quantum experiment 1 with 3 layers, (**d**) for quantum experiment 2 with 3 layers, (**e**) for quantum experiment 1 with 5 layers, and (**f**) for quantum experiment 2 with 5 layers. The x-axis shows the training epochs, while the y-axis shows the mean absolute error (standardized values). The black curve shows the test loss, while the magenta curve shows the validation loss.

**Figure 12:**
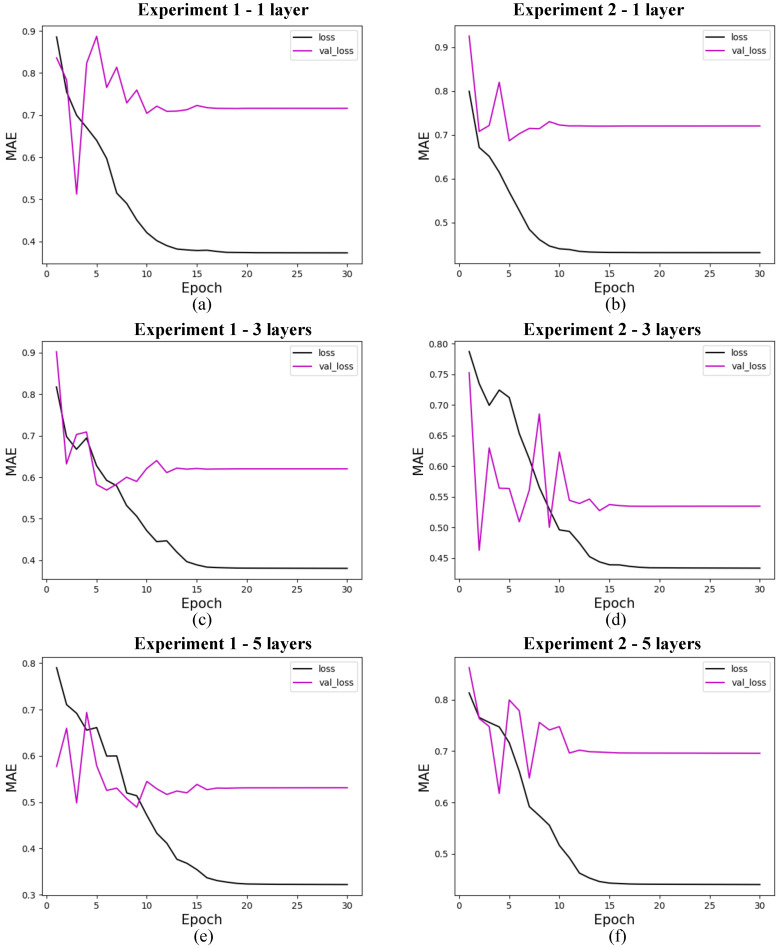
Loss for quantum models with 19 features. (**a**) shows the loss for quantum experiment 1 with 1 layer, (**b**) for quantum experiment 2 with 1 layer, (**c**) for quantum experiment 1 with 3 layers, (**d**) for quantum experiment 2 with 3 layers, (**e**) for quantum experiment 1 with 5 layers, and (**f**) for quantum experiment 2 with 5 layers. The x-axis shows the training epochs, while the y-axis shows the mean absolute error (standardized values). The black curve shows the test loss, while the magenta curve shows the validation loss.

The research on the convergence of quantum models showed that the results converge before the first 30 training epochs, so the 10 experiments used for the statistical analysis of each model were carried out using only 30 epochs. This decision was based on preliminary tests considering training with 1000 epochs, which showed rapid convergence of the models, as shown in Figure 9 for 4 features, with marginal or zero improvements in performance from that point onwards. Therefore, this approach optimizes the use of computational resources and avoids overtraining. In addition, although the models were simulated in a classical environment, quantum resources are currently scarce, so the predictive quality of the models linked to fast and stable convergence should be considered an advantage of these models. The convergence graphs of the models are shown in Figure 10, Figure 11 and Figure 12.

#### 5.5.1. Quantum Models

In this section, we present the performance of quantum models trained with 4, 8, and 19 input features. The loss curves are shown for different configurations of quantum circuits, varying the number of layers and experiment types. Figure 10 shows the results for models with 4 features, Figure 11 for models with 8 features, and Figure 12 for models with 19 features. Each figure illustrates the evolution of training loss over epochs, with comparisons between test and validation losses. The results provide insights into how circuit.

#### 5.5.2. Classical Models

**Figure 13:**
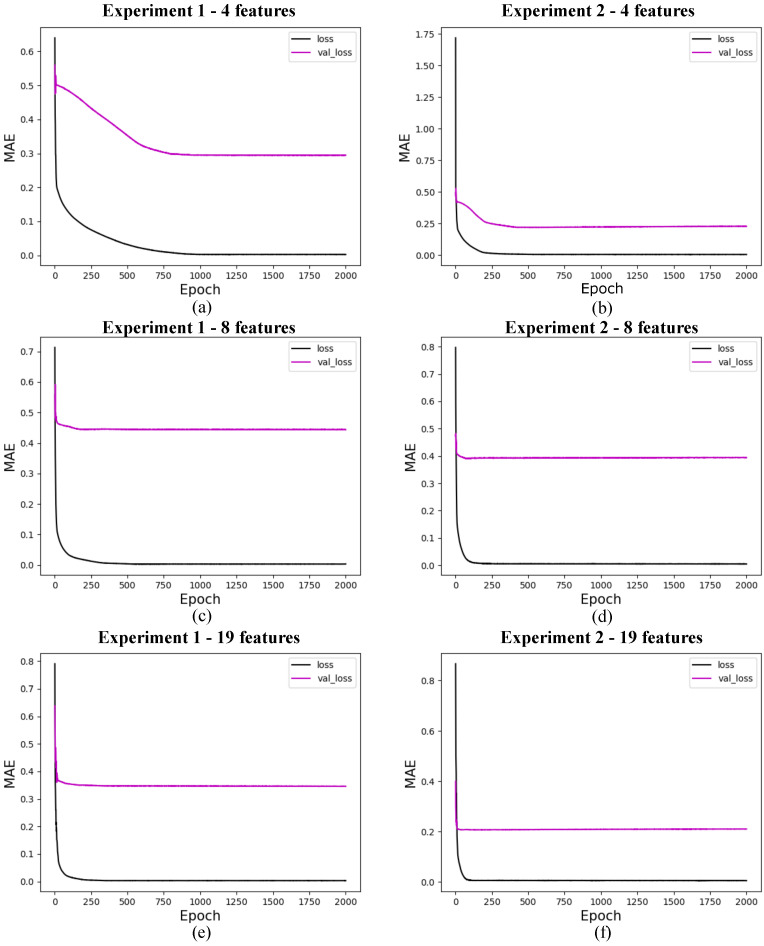
Loss for classical models. (**a**) shows the loss for classical experiment 1 with 4 features, (**b**) for classical experiment 2 with 4 features, (**c**) for classical experiment 1 with 8 features, (**d**) for classical experiment 2 with 8 features, (**e**) for quantum experiment 1 with 19 features, and (**f**) for classical experiment 2 with 19 features. The x-axis shows the training epochs, while the y-axis shows the mean absolute error (standardized values). The black curve shows the test loss, while the magenta curve shows the validation loss.

The validation loss of the classical model with 19 features, shown in Figure 13f, indicates that this model performs better than the other models, whether classical or quantum. However, the model’s performance with the test data, presented in Figure 8e–h, shows results that are not consistent with these findings. This discrepancy is due to the behavior of one of the features described earlier, where there was a significant and sudden increase in the data. The results that performed better with the test data, despite having a higher loss than that shown in Figure 13f, assigned a lower weight to the feature in question.

## 6. Conclusions

Here, a quantum neural network was utilized for the first time to solve the problem of predicting short-term demand for used vehicles. The tests were carried out on datasets with 4, 8, and 19 features. The results were compared with those obtained using a classical recurrent neural network, showing similarities of the models in terms of accuracy in the best case for each one, but with the quantum model using fewer features and parameters and converging in fewer epochs than the classical model. In addition, the quantum model showed less bias towards problematic features in the scenarios with the largest number of features considered. Thus, the results show evidence that quantum models can be excellent candidates for future implementation of this task in large-scale quantum computers. These results can possibly be extended to other predictions of interest to the financial sector, creating a new way of forecasting in the financial industry. These results could be better explored in subsequent stages by testing other quantum models, including quantum analogs to classical recurrent neural networks, and comparing the results with more robust variations of classical recurrent neural networks.

Code Availability: The code used in the experiments is available through the following link: https://github.com/morgoth00/quantum-demand-forecasting, accessed on 25 February 2025.

## Figures and Tables

**Table 1:** Best results of classical and quantum annual mean MAE.

Model		MAE
QNN	Experiment 1	682.52±284.14
	Experiment 2	785.98±192.21
RNN	Experiment 1	774.93±199.39
	Experiment 2	646.12±293.77

**Table 2:** Quantum model processing times.

		1 Layer	3 Layers	5 Layers
4 variáveis	Experiment 1	1 min 30	3 min	4 min
	Experiment 2	1 min 30	4 min	5 min
8 variáveis	Experiment 1	3 min	6 min	10 min
	Experiment 2	3 min	6.5 min	10 min
19 variáveis	Experiment 1	1 h	1 h 30	3 h 30
	Experiment 2	1 h	2 h 20	3 h 30

**Table 3:** Classical model processing times.

	Experiment 1	Experiment 2
4 features	13 min	32 min 30
8 features	14 min 30	36 min
19 features	14 min 30	36 min

**Figure A1:**
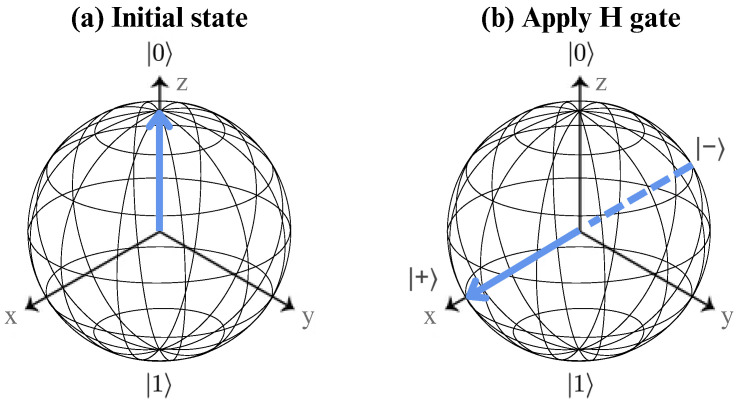
Bloch sphere representation of the *H* gate acting on a single qubit. (**a**) shows the initial qubit state, while (**b**) shows the state after applying this gate.

**Figure A2:**
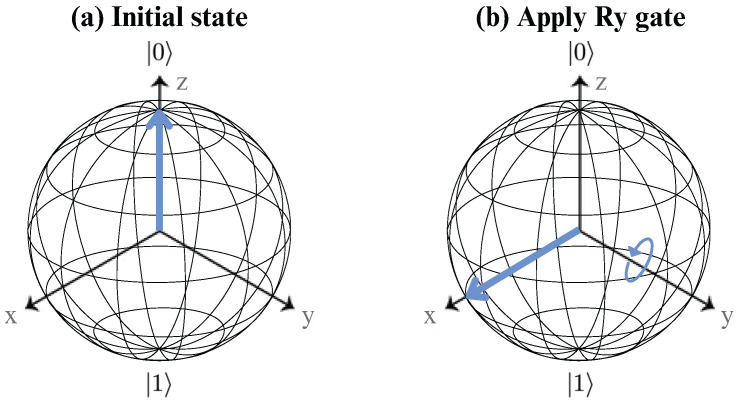
Bloch sphere representation of the $R_{y}\left(\theta \right)$ gate acting on a single qubit. (**a**) shows the initial qubit state, while (**b**) shows the state after applying this gate.

**Figure A3:**
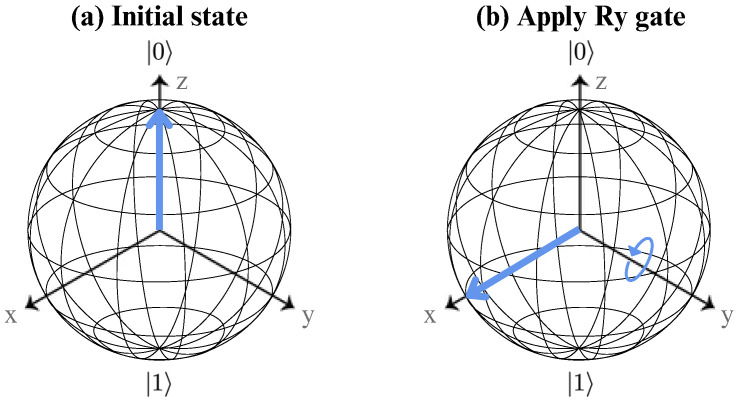
Bloch sphere representation of the $R_{x}\left(\theta \right)$ gate acting on a single qubit. (**a**) shows the initial qubit state, while (**b**) shows the state after applying this gate.

**Figure A4:**
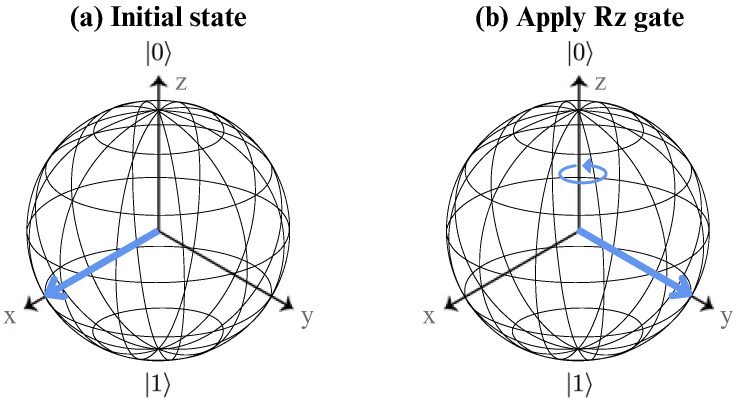
Bloch sphere representation of the $R_{z}\left(\theta \right)$ gate acting on a single qubit. (**a**) shows the initial qubit state, while (**b**) shows the state after applying this gate.

## Data Availability

The authors do not have permission to share data.

## Appendix A. The Algorithm

### Appendix A.1. Encoding

The first step of the algorithm involves encoding classical data into quantum bits. Initially, all qubits are prepared in a uniform superposition state using the Hadamard gate. This gate is a fundamental tool in quantum computing and is represented by the matrix shown in Equation (A1). The Hadamard gate ensures that each qubit has an equal probability of being measured in either the |0〉 or |1〉 state, thus enabling subsequent operations to be performed on superposed states. Figure A1 shows the visual representation of the action of this quantum logic gate on a qubit.

(A1)
$$H=\frac{1}{\sqrt{2}}\left[\begin{array}{cc} 1 & -1\\ 1 & 1 \end{array}\right].$$

However, at this stage, classical data have not yet been fully transformed into quantum states. The encoding process is achieved by applying an $R_{y}$ rotation gate to each qubit. The $R_{y}$ gate introduces a parameterized rotation around the y-axis of the Bloch sphere, effectively encoding numerical classical data into quantum amplitudes. This encoding step is pivotal, as it translates classical information into the quantum domain, enabling computations that exploit quantum mechanics. Equation (A2) provides the matrix representation of the $R_{y}$ gate, illustrating its dependence on the rotation angle parameter. Figure A2 shows a visual representation of the action of this quantum logic gate on a qubit.

(A2) $$R_{y}\left(\theta \right)=\left[\begin{array}{cc} \cos(\theta /2) & -\sin(\theta /2)\\ \sin(\theta /2) & \cos(\theta /2) \end{array}\right],$$

### Appendix A.2. Ansatz

The optimization process of the algorithm is implemented through an ansatz, which is a variational quantum circuit designed for specific problem-solving tasks. The ansatz is composed of various quantum gates, including the $R_{y}$ gate (introduced in Equation (A2)), as well as $R_{x}$, $R_{z}$, and CNOT gates. The $R_{x}$ and $R_{z}$ gates perform rotations around the X and Z axes of the Bloch sphere, respectively, allowing for adjustments to the phase and amplitude of the quantum states. Figure A3 and Figure A4 show the visual representation of the individual action of these gates on a qubit. The CNOT gate, a two-qubit entangling gate, introduces correlations between qubits, which are essential for harnessing the power of entanglement in quantum algorithms. The matrix representations of these gates, provided below, further detail their contributions to the ansatz structure.

(A3) $$R_{x}\left(\theta \right)=\left[\begin{array}{cc} \cos(\theta /2) & -i\sin(\theta /2)\\ -i\sin(\theta /2) & \cos(\theta /2) \end{array}\right],$$

(A4) $$R_{z}\left(\theta \right)=\left[\begin{array}{cc} e^{-i\theta /2} & 0\\ 0 & e^{i\theta /2} \end{array}\right].$$

(A5) $$CNOT=\left[\begin{array}{cccc} 1 & 0 & 0 & 0\\ 0 & 1 & 0 & 0\\ 0 & 0 & 0 & 1\\ 0 & 0 & 1 & 0 \end{array}\right]$$

### Appendix A.3. Measurements

Measurements at the end of the quantum circuit are carried out by projecting the states of the qubits onto the Z basis, effectively collapsing the superposition states into classical bits. This process is probabilistic, as the outcome depends on the quantum state amplitudes defined during the encoding and optimization steps. To ensure reliable results, a statistically significant number of measurements must be performed, with outcomes aggregated to determine the probabilities of each result. This measurement step is critical for extracting meaningful information from quantum computations.

### Appendix A.4. Optimization

The optimization step is performed classically. In this step, the angles of the gates in the ansatz are adjusted by a classical optimizer. This optimizer iteratively refines the gate parameters to minimize a cost function that is typically related to the target problem being solved. Once the optimizer determines new optimal angles, these updated angles are applied to a new quantum circuit. The circuit retains the same structure as the previous one but incorporates the new angles into the ansatz. This iterative process continues until convergence is achieved or a predefined criterion is met, ensuring an efficient approach to solving the problem at hand.

## Appendix B. Standard Deviation

The monthly standard deviation obtained in each experiment is presented in this section. Appendix B.1 shows the deviations obtained in the quantum experiments, while Appendix B.2 shows the deviations obtained in the classical experiments.

### Appendix B.1. Quantum Experiments

Table A1, Table A2 and Table A3 show the standard deviation obtained in the quantum experiments carried out with 4, 8, and 19 features, respectively. The columns of the tables represent each experiment and the number of layers used, while the rows represent the deviations in each month. The last two rows represent the mean and median deviations over the 12-month period.

**Table A1:** Monthly standard deviation for quantum experiments with 4 features. The columns represent each experiment with a number of 1, 3, and 5 variational layers, and the lines represent the months. The last two lines show the 12-month mean and median of the mean absolute error.

	Experiment 1			Experiment 2		
	1 Layer	3 Layers	5 Layers	1 Layer	3 Layers	5 Layers
Month 1	175.8777	449.1271	579.0006	292.4347	781.3115	945.6261
Month 2	160.8550	454.754	541.9076	311.3989	697.3065	928.2651
Month 3	243.3442	517.2781	590.0444	324.5650	812.6765	100.6944
Month 4	348.6137	574.0108	598.8600	410.6001	871.5712	1111.7944
Month 5	384.9921	710.9180	472.9006	355.1420	780.3099	795.7520
Month 6	443.4344	770.3615	422.4470	326.7457	730.8906	498.5881
Month 7	345.1314	893.0815	746.0978	629.6234	858.7378	506.8464
Month 8	815.3586	990.5417	737.8833	707.0781	1085.2201	506.1400
Month 9	543.9914	982.4916	804.3594	871.3573	1055.3599	458.0408
Month 10	643.1245	810.9172	597.7432	578.6797	931.9728	502.1143
Month 11	738.7717	658.1062	395.4243	347.0588	1020.1457	794.2041
Month 12	905.4925	695.5312	451.0824	302.2530	1077.0132	679.5169
Mean	479.0823	708.8950	578.1459	454.7447	891.8763	727.3819
Median	414.2133	703.2246	584.5225	351.1004	865.1545	736.8605

**Table A2:** Monthly standard deviation for quantum experiments with 8 features. The columns represent each experiment with a number of 1, 3, and 5 variational layers, and the lines represent the months. The last two lines show the 12-month mean and median of the mean absolute error.

	Experiment 1			Experiment 2		
	1 Layer	3 Layers	5 Layers	1 Layer	3 Layers	5 Layers
Month 1	160.8833	252.3982	326.4278	121.1870	540.7551	466.1218
Month 2	389.2180	321.1851	321.9523	115.6250	544.9329	578.9061
Month 3	199.1200	407.9355	427.0799	116.9056	628.8543	312.7844
Month 4	317.9407	397.5779	462.1943	276.6656	595.7785	338.2273
Month 5	406.4636	597.9564	480.5169	345.8178	437.5681	255.3891
Month 6	710.3341	482.3563	550.8562	235.6304	739.2531	322.0360
Month 7	557.0957	722.5253	642.4665	575.8245	602.2518	353.3172
Month 8	1059.4827	621.9092	885.9215	693.9122	616.6715	352.7128
Month 9	758.2744	793.3121	874.5666	801.1654	976.0197	579.2410
Month 10	841.0811	915.8552	820.2909	697.0682	655.6456	514.6416
Month 11	793.1211	887.0502	612.1734	269.6858	615.5451	507.4711
Month 12	1245.5292	529.3304	646.7094	483.5458	799.0230	362.0224
Mean	619.8787	577.4493	587.5963	394.4194	646.0249	411.9059
Median	633.7149	563.6434	581.5148	311.2417	616.1083	357.6698

**Table A3:** Monthly standard deviation for quantum experiments with 19 features. The columns represent each experiment with a number of 1, 3, and 5 variational layers, and the lines represent the months. The last two lines show the 12-month mean and median of the mean absolute error.

	Experiment 1			Experiment 2		
	1 Layer	3 Layers	5 Layers	1 Layer	3 Layers	5 Layers
Month 1	516.7164	494.9935	578.7277	355.9007	427.1650	521.0726
Month 2	572.1335	685.2427	280.9679	145.9410	421.7772	693.0295
Month 3	586.1252	738.5064	532.6228	675.5994	658.4324	688.9236
Month 4	598.8343	669.0032	586.9466	590.2582	758.9672	748.5518
Month 5	639.3816	539.5154	444.1018	266.6220	437.0098	685.1440
Month 6	1091.7456	705.1529	436.6959	519.9328	534.6220	579.4321
Month 7	999.3549	665.8485	439.2488	413.0658	689.7512	570.5552
Month 8	647.7254	797.2639	559.3305	932.4816	570.7285	670.7190
Month 9	967.2389	707.2880	587.0741	1128.5844	684.2530	737.8799
Month 10	1389.6125	641.6429	565.2284	1301.0933	705.1833	659.6317
Month 11	953.2446	811.4675	466.9461	920.2317	748.9888	479.6019
Month 12	995.1870	863.9411	424.4394	959.6248	798.4880	565.5388
Mean	829.7750	693.3222	491.8608	648.1113	619.6139	633.3400
Median	800.4850	695.1979	499.7845	632.9288	671.3427	665.1754

### Appendix B.2. Classical Experiments

Table A4 shows the standard deviation obtained in the classical experiments carried out with 4, 8, and 19 features. The columns of the table represent each experiment and the number of features used, while the rows represent the deviations in each month. The last two rows represent the average and median deviations over the 12-month period.

**Table A4:** Monthly standard deviation for classical experiments with 4, 8, and 19 features. The columns represent each experiment with a number of 4, 8, and 19 features, and the lines represent the months. The last two lines show the 12-month mean and median of the mean absolute error.

	Classical Experiment 1			Classical Experiment 2		
	4 Features	8 Features	19 Features	4 Features	8 Features	19 Features
Month 1	308.17	515.30	983.76	99.29	189.26	263.49
Month 2	354.86	592.63	928.49	258.99	240.52	368.53
Month 3	528.00	597.08	684.88	221.78	155.91	158.13
Month 4	495.66	797.64	817.02	321.39	252.95	190.64
Month 5	472.31	703.64	676.05	333.65	171.95	136.29
Month 6	552.07	611.75	748.18	287.49	197.82	353.89
Month 7	484.05	613.97	494.19	346.032	230.98	327.44
Month 8	521.11	929.49	218,484.99	492.37	213.54	66,181.53
Month 9	766.99	729.89	217,021.29	573.75	204.83	87,269.59
Month 10	809.57	621.17	224,172.77	623.91	312.91	94,615.78
Month 11	797.50	760.97	231,876.29	322.52	218.21	63,811.92
Month 12	697.86	751.18	260,196.92	385.86	154.92	55,942.54
Mean	565.68	685.39	96,423.73	355.59	211,598	30,801.65
Median	524.55	662.40	956.13	328.09	209.19	361.21

## Appendix C. Mean Absolute Error

The mean absolute error obtained in each experiment is presented in this section. Appendix C.1 presents the errors obtained in the quantum experiments, while Appendix C.2 presents the errors obtained in the classical experiments.

### Appendix C.1. Quantum Experiments

Table A5, Table A6 and Table A7 show the mean absolute error obtained in the quantum experiments carried out with 4, 8, and 19 features, respectively. The columns of the tables represent each experiment and the number of layers used, while the rows represent the errors in each month. The last row represents the average of the mean absolute errors over the 12-month period.

**Table A5:** Monthly mean absolute error for quantum experiments with 4 features. The columns represent each experiment with a number of 1, 3, and 5 variational layers, and the lines represent the months. The last line shows the 12-month mean of the mean absolute error.

	Experiment 1			Experiment 2		
	1 Layer	3 Layers	5 Layers	1 Layer	3 Layers	5 Layers
Month 1	650.18	401.92	530.33	792.33	1463.55	871.18
Month 2	1175.84	536.48	646.12	805.95	1469.63	951.18
Month 3	635.44	485.10	496.18	1117.42	1479.38	944.70
Month 4	299.90	453.46	513.00	804.10	1120.72	929.66
Month 5	334.44	707.43	858.72	1560.78	738.46	673.11
Month 6	493.43	658.21	842.96	2249.48	698.33	411.97
Month 7	733.64	886.78	717.68	2770.17	912.10	626.01
Month 8	1144.93	1027.13	827.18	2888.23	1013.24	867.35
Month 9	566.27	1390.83	1297.83	3154.11	885.93	522.42
Month 10	577.18	898.56	698.99	2561.83	1130.34	742.62
Month 11	596.82	1036.78	1036.90	1979.68	821.36	849.04
Month 12	982.13	792.25	641.45	2048.10	1066.48	1042.62
Mean	682.52 ± 284.14	772.91 ± 292.49	758.94 ± 234.12	1894.35 ± 865.87	1066.63 ± 279.64	785.98 ± 192.21

**Table A6:** Monthly mean absolute error for quantum experiments with 8 features. The columns represent each experiment with a number of 1, 3, and 5 variational layers, and the lines represent the months. The last line shows the 12-month mean of the mean absolute error.

	Experiment 1			Experiment 2		
	1 Layer	3 Layers	5 Layers	1 Layer	3 Layers	5 Layers
Month 1	512.12	732.27	401.66	1097.63	1217.56	951.43
Month 2	616.63	794.92	530.46	1091.54	1366.40	1290.20
Month 3	410.03	795.86	393.68	1316.14	1277.38	1179.36
Month 4	346.10	439.18	431.12	724.89	835.73	683.89
Month 5	398.91	585.06	832.43	1327.13	647.49	441.65
Month 6	850.79	427.59	632.40	2452.52	1028.89	1035.87
Month 7	760.58	767.84	921.27	2418.72	1075.02	993.72
Month 8	1008.91	782.52	1008.66	2852.20	938.75	1180.06
Month 9	705.20	1388.40	1597.51	2948.62	884.34	941.42
Month 10	692.89	952.77	1075.67	1764.08	916.54	935.58
Month 11	806.42	1036.14	1186.70	1923.66	684.19	899.97
Month 12	1408.56	596.32	881.29	1709.09	1053.04	1236.42
Mean	709.76 ± 297.93	774.90 ± 266.34	824.40 ± 366.91	1802.19 ± 730.42	993.78 ± 222.06	980.79 ± 239.93

**Table A7:** Monthly mean absolute error for quantum experiments with 19 features. The columns represent each experiment with a number of 1, 3, and 5 variational layers, and the lines represent the months. The last line shows the 12-month mean of the mean absolute error.

	Experiment 1			Experiment 2		
	1 Layer	3 Layers	5 Layers	1 Layer	3 Layers	5 Layers
Month 1	1290.76	1271.09	1184.78	2580.36	1465.18	1669.35
Month 2	1138.65	1341.46	1113.86	2454.71	1664.07	1878.59
Month 3	1012.17	864.44	852.39	1860.12	1122.22	1412.05
Month 4	990.79	990.92	1030.96	1660.29	801.17	1150.94
Month 5	522.95	651.76	533.07	1597.31	987.06	1119.59
Month 6	935.99	1132.94	1024.33	1732.11	785.72	775.02
Month 7	835.87	1570.97	1529.91	2496.80	1508.08	1190.74
Month 8	525.35	1240.00	1177.67	758.83	967.37	1232.49
Month 9	1090.46	710.54	629.81	1014.02	592.40	564.18
Month 10	2013.58	1264.20	1509.81	1208.45	623.42	892.39
Month 11	1442.46	638.46	791.39	967.58	552.07	365.82
Month 12	1899.93	977.43	1238.75	1241.23	879.85	647.64
Mean	1141.58 ± 465.98	1054.52 ± 298.52	1051.39 ± 311.39	1630.98 ± 625.66	995.71 ± 374.16	1074.90 ± 450.09

### Appendix C.2. Classical Experiments

Table A8 shows the mean absolute error obtained in the classical experiments carried out with 4, 8, and 19 features. The columns of the table represent each experiment and the number of features used, while the rows represent the error obtained in each month. The last two rows represent the average and deviations over the 12-month period.

**Table A8:** Monthly mean absolute error for classical experiments with 4, 8, and 19 features. The columns represent each experiment with a number of 4, 8, and 19 features, and the lines represent the months. The last line shows the 12-month mean of the mean absolute error.

	Classical Experiment 1			Classical Experiment 2		
	4 Features	8 Features	19 Features	4 Features	8 Features	19 Features
Month 1	1080.65	1214.68	1083.70	852.70	806.36	331.93
Month 2	772.14	1195.05	1083.74	549.44	886.17	497.79
Month 3	934.07	1513.97	1422.53	821.23	1255.03	700.34
Month 4	658.06	1244.07	806.78	332.87	781.47	229.77
Month 5	549.08	1104.81	796.94	272.64	584.70	97.59
Month 6	725.33	1006.55	894.82	380.18	1035.19	289.33
Month 7	909.29	1425.20	535.88	539.62	1021.39	262.57
Month 8	477.09	663.75	191,450.99	286.32	357.82	83,613.94
Month 9	967.82	663.77	207,996.69	1005.15	199.17	178,754.93
Month 10	802.83	645.03	232,189.14	850.08	302.93	129,657.59
Month 11	933.40	673.35	211,070.68	1136.52	557.77	175,224.64
Month 12	489.41	615.22	236,433.09	726.71	411.61	170,439.79
Mean	774.93 ± 199.39	997.12 ± 331.18	90,480.42 ± 111,210.54	646.12 ± 293.77	683.30 ± 332.37	79,695.14 ± 61,675.02

## Appendix D. Mean Absolute Percentage Error

The mean absolute percentage error obtained in each experiment is presented in this section. Appendix D.1 presents the errors obtained in the quantum experiments, while Appendix D.2 presents the errors obtained in the classical experiments.

### Appendix D.1. Quantum Experiments

**Table A9:** Monthly mean absolute percentage error for quantum experiments with 4 features. The columns represent each experiment with a number of 1, 3, and 5 variational layers, and the lines represent the months. The last line shows the 12-month mean of the mean absolute percentage error.

	Experiment 1			Experiment 2		
	1 Layer	3 Layers	5 Layers	1 Layer	3 Layers	5 Layers
Month 1	5.78	3.57	4.71	7.05	13.02	7.75
Month 2	10.58	4.83	5.82	7.25	13.23	8.56
Month 3	5.68	4.34	4.43	9.99	13.22	8.44
Month 4	2.57	3.88	4.39	6.88	9.59	7.96
Month 5	2.77	5.87	7.12	12.95	6.13	5.58
Month 6	4.13	5.51	7.06	18.83	5.85	3.45
Month 7	6.45	7.79	6.30	24.34	8.01	5.50
Month 8	10.04	9.01	7.25	25.33	8.89	7.61
Month 9	4.79	11.77	10.98	26.69	7.50	4.42
Month 10	5.02	7.82	6.08	22.30	9.84	6.46
Month 11	4.99	8.66	8.66	16.54	6.86	7.09
Month 12	8.49	6.85	5.55	17.71	9.22	9.01
Mean	5.94 ± 2.22	6.65 ± 2.34	6.53 ± 1.88	16.32 ± 6.93	9.28 ± 2.22	6.82 ± 1.53

**Table A10:** Monthly mean absolute percentage error for quantum experiments with 8 features. The columns represent each experiment with a number of 1, 3, and 5 variational layers, and the lines represent the months. The last line shows the 12-month mean of the mean absolute percentage error.

	Experiment 1			Experiment 2		
	1 Layer	3 Layers	5 Layers	1 Layer	3 Layers	5 Layers
Month 1	4.56	6.51	3.57	9.76	10.83	8.46
Month 2	5.55	7.16	4.77	9.82	12.30	11.61
Month 3	3.66	7.11	3.52	11.76	11.42	10.54
Month 4	2.96	3.76	3.69	6.21	7.15	5.85
Month 5	3.31	4.85	6.91	11.01	5.37	3.66
Month 6	7.12	3.58	5.29	20.53	8.61	8.67
Month 7	6.68	6.75	8.09	21.25	9.44	8.73
Month 8	8.85	6.86	8.84	25.01	8.23	10.35
Month 9	5.97	11.75	13.52	24.95	7.48	7.97
Month 10	6.03	8.29	9.36	15.35	7.98	8.14
Month 11	6.74	8.66	9.91	16.07	5.72	7.52
Month 12	12.18	5.16	7.62	14.78	9.11	10.69
Mean	6.13 ± 2.38	6.70 ± 2.06	7.09 ± 2.81	15.54 ± 5.78	8.64 ± 1.87	8.52 ± 2.13

**Table A11:** Monthly mean absolute percentage error for quantum experiments with 19 features. The columns represent each experiment with a number of 1, 3, and 5 variational layers, and the lines represent the months. The last line shows the 12-month mean of the mean absolute percentage error.

	Experiment 1			Experiment 2		
	1 Layer	3 Layers	5 Layers	1 Layer	3 Layers	5 Layers
Month 1	11.48	11.31	10.54	22.95	13.03	14.85
Month 2	10.25	12.07	10.03	22.10	14.98	16.91
Month 3	9.05	7.73	7.62	16.63	10.03	12.62
Month 4	8.48	8.48	8.82	14.21	6.86	9.85
Month 5	4.34	5.41	4.42	13.25	8.19	9.29
Month 6	7.84	9.48	8.57	14.50	6.58	6.49
Month 7	7.34	13.80	13.44	21.93	13.25	10.46
Month 8	4.60	10.87	10.33	6.66	8.48	10.81
Month 9	9.23	6.01	5.33	8.58	5.01	4.77
Month 10	17.53	11.00	13.14	10.52	5.43	7.77
Month 11	12.05	5.33	6.61	8.08	4.61	3.06
Month 12	16.43	8.45	10.71	10.73	7.61	5.60
Mean	9.88 ± 3.71	9.16 ± 2.53	9.13 ± 2.74	14.18 ± 4.74	8.67 ± 3.27	9.37 ± 3.86

### Appendix D.2. Classical Experiments

**Table A12:** Monthly mean absolute percentage error for classical experiments with 4, 8, and features. The columns represent each experiment with a number of 4, 8, and 19 features, and the lines represent the months. The last line shows the 12-month mean of the mean absolute percentage error.

	Classical Experiment 1			Classical Experiment 2		
	4 Features	8 Features	19 Features	4 Features	8 Features	19 Features
Month 1	9.61	10.80	9.64	7.58	7.17	2.95
Month 2	6.95	10.76	9.76	4.95	7.98	4.48
Month 3	8.35	13.53	12.71	7.34	11.22	6.26
Month 4	5.63	10.65	6.91	2.85	6.69	1.97
Month 5	4.56	9.17	6.61	2.26	4.85	0.81
Month 6	6.07	8.43	7.49	3.18	8.67	2.42
Month 7	7.99	12.52	4.71	4.74	8.97	2.30
Month 8	4.18	5.82	1679.05	2.51	3.14	733.31
Month 9	8.19	5.62	1759.88	8.50	1.68	1512.47
Month 10	6.99	5.61	2021.14	7.40	2.64	1128.63
Month 11	7.80	5.62	1763.24	9.50	4.66	1463.79
Month 12	4.23	5.32	2044.47	6.28	3.56	1473.82
Mean	6.71 ± 1.61	8.65 ± 2.74	777.13 ± 841.90	5.59 ± 2.08	5.93 ± 2.56	527.77 ± 599.01

## Appendix E. Mean Squared Error

### Appendix E.1. Quantum Experiments

**Table A13:** Monthly mean squared error for quantum experiments with 4 features. The columns represent each experiment with a number of 1, 3, and 5 variational layers, and the lines represent the months. The last line shows the 12-month mean of the mean squared error.

	Experiment 1			Experiment 2		
	1 Layer	3 Layers	5 Layers	1 Layer	3 Layers	5 Layers
Month 1	$4.51\times 10^{5}$	$2.39\times 10^{5}$	$3.69\times 10^{5}$	$7.05\times 10^{5}$	$2.45\times 10^{6}$	$1.21\times 10^{6}$
Month 2	$1.41\times 10^{6}$	$4.37\times 10^{5}$	$5.26\times 10^{5}$	$7.37\times 10^{5}$	$2.53\times 10^{6}$	$1.32\times 10^{6}$
Month 3	$4.57\times 10^{5}$	$2.78\times 10^{5}$	$3.42\times 10^{5}$	$1.34\times 10^{6}$	$2.54\times 10^{6}$	$1.39\times 10^{6}$
Month 4	$1.53\times 10^{5}$	$4.73\times 10^{5}$	$5.86\times 10^{5}$	$7.98\times 10^{5}$	$1.53\times 10^{6}$	$1.26\times 10^{6}$
Month 5	$1.35\times 10^{5}$	$9.53\times 10^{5}$	$9.39\times 10^{5}$	$2.55\times 10^{6}$	$7.09\times 10^{5}$	$5.80\times 10^{5}$
Month 6	$2.99\times 10^{5}$	$9.33\times 10^{5}$	$8.71\times 10^{5}$	$5.16\times 10^{6}$	$6.20\times 10^{5}$	$2.24\times 10^{5}$
Month 7	$6.68\times 10^{5}$	$1.19\times 10^{6}$	$8.19\times 10^{5}$	$7.75\times 10^{6}$	$1.12\times 10^{6}$	$5.96\times 10^{5}$
Month 8	$1.74\times 10^{6}$	$1.61\times 10^{6}$	$9.34\times 10^{5}$	$8.47\times 10^{6}$	$1.39\times 10^{6}$	$9.68\times 10^{5}$
Month 9	$4.16\times 10^{5}$	$2.68\times 10^{6}$	$2.01\times 10^{6}$	$1.00\times 10^{7}$	$1.07\times 10^{6}$	$4.14\times 10^{5}$
Month 10	$4.72\times 10^{5}$	$1.06\times 10^{6}$	$6.28\times 10^{5}$	$6.62\times 10^{6}$	$1.57\times 10^{6}$	$7.37\times 10^{5}$
Month 11	$5.18\times 10^{5}$	$1.46\times 10^{6}$	$1.22\times 10^{6}$	$4.03\times 10^{6}$	$9.90\times 10^{5}$	$1.01\times 10^{6}$
Month 12	$1.35\times 10^{6}$	$9.83\times 10^{5}$	$5.95\times 10^{5}$	$4.28\times 10^{6}$	$1.52\times 10^{6}$	$1.37\times 10^{6}$
Mean	$6.72\times 10^{5}\pm 4.00\times 10^{5}$	$1.02\times 10^{6}\pm 6.88\times 10^{5}$	$8.20\times 10^{5}\pm 4.98\times 10^{5}$	$4.37\times 10^{6}\pm 2.36\times 10^{6}$	$1.50\times 10^{6}\pm 6.52\times 10^{5}$	$9.23\times 10^{5}\pm 4.34\times 10^{5}$

**Table A14:** Monthly mean squared error for quantum experiments with 8 features. The columns represent each experiment with a number of 1, 3, and 5 variational layers, and the lines represent the months. The last line shows the 12-month mean of the mean squared error.

	Experiment 1			Experiment 2		
	1 Layer	3 Layers	5 Layers	1 Layer	3 Layers	5 Layers
Month 1	$2.86\times 10^{5}$	$5.94\times 10^{5}$	$2.35\times 10^{5}$	$1.22\times 10^{6}$	$1.75\times 10^{6}$	$1.10\times 10^{6}$
Month 2	$4.47\times 10^{5}$	$7.25\times 10^{5}$	$3.75\times 10^{5}$	$1.20\times 10^{6}$	$2.13\times 10^{6}$	$1.97\times 10^{6}$
Month 3	$2.04\times 10^{5}$	$7.83\times 10^{5}$	$2.57\times 10^{5}$	$1.74\times 10^{6}$	$1.99\times 10^{6}$	$1.48\times 10^{6}$
Month 4	$1.70\times 10^{5}$	$2.67\times 10^{5}$	$2.50\times 10^{5}$	$5.94\times 10^{5}$	$8.84\times 10^{5}$	$5.71\times 10^{5}$
Month 5	$2.42\times 10^{5}$	$4.18\times 10^{5}$	$8.87\times 10^{5}$	$1.87\times 10^{6}$	$5.92\times 10^{5}$	$2.54\times 10^{5}$
Month 6	$1.06\times 10^{6}$	$3.28\times 10^{5}$	$5.46\times 10^{5}$	$6.06\times 10^{6}$	$1.31\times 10^{6}$	$1.17\times 10^{6}$
Month 7	$8.11\times 10^{5}$	$6.86\times 10^{5}$	$9.48\times 10^{5}$	$5.97\times 10^{6}$	$1.45\times 10^{6}$	$1.10\times 10^{6}$
Month 8	$1.61\times 10^{6}$	$7.27\times 10^{5}$	$1.38\times 10^{6}$	$8.32\times 10^{6}$	$1.21\times 10^{6}$	$1.52\times 10^{6}$
Month 9	$7.26\times 10^{5}$	$2.41\times 10^{6}$	$2.99\times 10^{6}$	$8.78\times 10^{6}$	$1.03\times 10^{6}$	$1.11\times 10^{6}$
Month 10	$6.80\times 10^{5}$	$1.37\times 10^{6}$	$1.57\times 10^{6}$	$3.49\times 10^{6}$	$1.15\times 10^{6}$	$1.11\times 10^{6}$
Month 11	$9.36\times 10^{5}$	$1.46\times 10^{6}$	$1.75\times 10^{6}$	$3.77\times 10^{6}$	$6.26\times 10^{5}$	$1.04\times 10^{6}$
Month 12	$3.08\times 10^{6}$	$4.89\times 10^{5}$	$1.15\times 10^{6}$	$3.13\times 10^{6}$	$1.49\times 10^{6}$	$1.65\times 10^{6}$
Mean	$8.54\times 10^{5}\pm 7.39\times 10^{5}$	$8.55\times 10^{5}\pm 5.70\times 10^{5}$	$1.03\times 10^{6}\pm 7.15\times 10^{5}$	$3.85\times 10^{6}\pm 2.07\times 10^{6}$	$1.30\times 10^{6}\pm 5.56\times 10^{5}$	$1.17\times 10^{6}\pm 4.58\times 10^{5}$

**Table A15:** Monthly mean squared error for quantum experiments with 19 features. The columns represent each experiment with a number of 1, 3, and 5 variational layers, and the lines represent the months. The last line shows the 12-month mean of the mean squared error.

	Experiment 1			Experiment 2		
	1 Layer	3 Layers	5 Layers	1 Layer	3 Layers	5 Layers
Month 1	$1.91\times 10^{6}$	$1.84\times 10^{6}$	$1.71\times 10^{6}$	$6.77\times 10^{6}$	$2.31\times 10^{6}$	$3.03\times 10^{6}$
Month 2	$1.59\times 10^{6}$	$2.22\times 10^{6}$	$1.31\times 10^{6}$	$6.04\times 10^{6}$	$2.93\times 10^{6}$	$3.96\times 10^{6}$
Month 3	$1.33\times 10^{6}$	$1.20\times 10^{6}$	$9.82\times 10^{5}$	$3.87\times 10^{6}$	$1.65\times 10^{6}$	$2.42\times 10^{6}$
Month 4	$1.30\times 10^{6}$	$1.38\times 10^{6}$	$1.28\times 10^{6}$	$3.07\times 10^{6}$	$1.08\times 10^{6}$	$1.75\times 10^{6}$
Month 5	$4.01\times 10^{5}$	$6.40\times 10^{5}$	$4.26\times 10^{5}$	$2.62\times 10^{6}$	$1.15\times 10^{6}$	$1.68\times 10^{6}$
Month 6	$1.39\times 10^{6}$	$1.73\times 10^{6}$	$1.22\times 10^{6}$	$3.24\times 10^{6}$	$8.46\times 10^{5}$	$9.03\times 10^{5}$
Month 7	$9.67\times 10^{5}$	$2.93\times 10^{6}$	$2.48\times 10^{6}$	$6.36\times 10^{6}$	$2.65\times 10^{6}$	$1.70\times 10^{6}$
Month 8	$3.77\times 10^{5}$	$2.02\times 10^{6}$	$1.61\times 10^{6}$	$8.48\times 10^{5}$	$1.14\times 10^{6}$	$1.91\times 10^{6}$
Month 9	$1.72\times 10^{6}$	$7.92\times 10^{5}$	$5.83\times 10^{5}$	$1.37\times 10^{6}$	$4.76\times 10^{5}$	$6.51\times 10^{5}$
Month 10	$5.15\times 10^{6}$	$1.95\times 10^{6}$	$2.54\times 10^{6}$	$1.75\times 10^{6}$	$5.42\times 10^{5}$	$1.15\times 10^{6}$
Month 11	$2.90\times 10^{6}$	$7.33\times 10^{5}$	$8.23\times 10^{5}$	$1.19\times 10^{6}$	$5.05\times 10^{5}$	$2.53\times 10^{5}$
Month 12	$4.50\times 10^{6}$	$1.57\times 10^{6}$	$1.70\times 10^{6}$	$1.89\times 10^{6}$	$8.39\times 10^{5}$	$6.90\times 10^{5}$
Mean	$1.96\times 10^{6}\pm 1.29\times 10^{6}$	$1.58\times 10^{6}\pm 6.39\times 10^{5}$	$1.39\times 10^{6}\pm 6.87\times 10^{5}$	$3.25\times 10^{6}\pm 1.91\times 10^{6}$	$1.34\times 10^{6}\pm 7.34\times 10^{5}$	$1.67\times 10^{6}\pm 1.04\times 10^{6}$

### Appendix E.2. Classical Experiments

**Table A16:** Monthly mean squared error for classical experiments with 4, 8 and 19 features. The columns represent each experiment with a number of 4, 8 and 19 features, and the lines represent the months. The last line shows the 12-month mean of the mean squared error.

	Experiment 1			Experiment 2		
	4 Features	8 Features	19 Features	4 Features	8 Features	19 Features
Month 1	$1.25\times 10^{6}$	$1.71\times 10^{6}$	$1.90\times 10^{6}$	$7.36\times 10^{5}$	$6.82\times 10^{5}$	$1.45\times 10^{5}$
Month 2	$7.10\times 10^{5}$	$1.74\times 10^{6}$	$1.95\times 10^{6}$	$3.62\times 10^{5}$	$8.37\times 10^{5}$	$3.63\times 10^{5}$
Month 3	$1.12\times 10^{6}$	$2.61\times 10^{6}$	$2.45\times 10^{6}$	$7.19\times 10^{5}$	$1.60\times 10^{6}$	$5.13\times 10^{5}$
Month 4	$5.92\times 10^{5}$	$2.06\times 10^{6}$	$1.11\times 10^{6}$	$1.70\times 10^{5}$	$6.68\times 10^{5}$	$8.55\times 10^{4}$
Month 5	$4.35\times 10^{5}$	$1.67\times 10^{6}$	$8.97\times 10^{5}$	$1.03\times 10^{5}$	$3.68\times 10^{5}$	$1.77\times 10^{4}$
Month 6	$7.87\times 10^{5}$	$1.35\times 10^{6}$	$1.18\times 10^{6}$	$1.85\times 10^{5}$	$1.11\times 10^{6}$	$1.72\times 10^{5}$
Month 7	$1.06\times 10^{6}$	$2.37\times 10^{6}$	$4.64\times 10^{5}$	$3.56\times 10^{5}$	$1.08\times 10^{6}$	$1.02\times 10^{5}$
Month 8	$3.90\times 10^{5}$	$6.08\times 10^{5}$	$5.00\times 10^{10}$	$1.53\times 10^{5}$	$1.70\times 10^{5}$	$8.06\times 10^{9}$
Month 9	$1.15\times 10^{6}$	$6.14\times 10^{5}$	$6.98\times 10^{10}$	$1.10\times 10^{6}$	$5.65\times 10^{4}$	$3.63\times 10^{10}$
Month 10	$9.54\times 10^{5}$	$6.45\times 10^{5}$	$9.01\times 10^{10}$	$8.30\times 10^{5}$	$1.08\times 10^{5}$	$2.40\times 10^{10}$
Month 11	$1.23\times 10^{6}$	$7.69\times 10^{5}$	$8.20\times 10^{10}$	$1.39\times 10^{6}$	$3.54\times 10^{5}$	$3.44\times 10^{10}$
Month 12	$4.39\times 10^{5}$	$5.14\times 10^{5}$	$8.58\times 10^{10}$	$6.62\times 10^{5}$	$1.91\times 10^{5}$	$3.19\times 10^{10}$
Mean	$8.44\times 10^{5}\pm 3.12\times 10^{5}$	$1.39\times 10^{6}\pm 7.14\times 10^{5}$	$3.15\times 10^{10}\pm 3.84\times 10^{10}$	$5.64\times 10^{5}\pm 3.95\times 10^{5}$	$6.02\times 10^{5}\pm 4.62\times 10^{5}$	$1.12\times 10^{10}\pm 1.49\times 10^{10}$
